# Supplementary figures and images for: Die-off of plant pathogenic bacteria in tile drainage and anoxic water from a managed aquifer recharge site
Source: PLoS One. 2021 May 5;16(5):e0250338. doi: 10.1371/journal.pone.0250338 (PMC8099070; doi:10.1371/journal.pone.0250338)

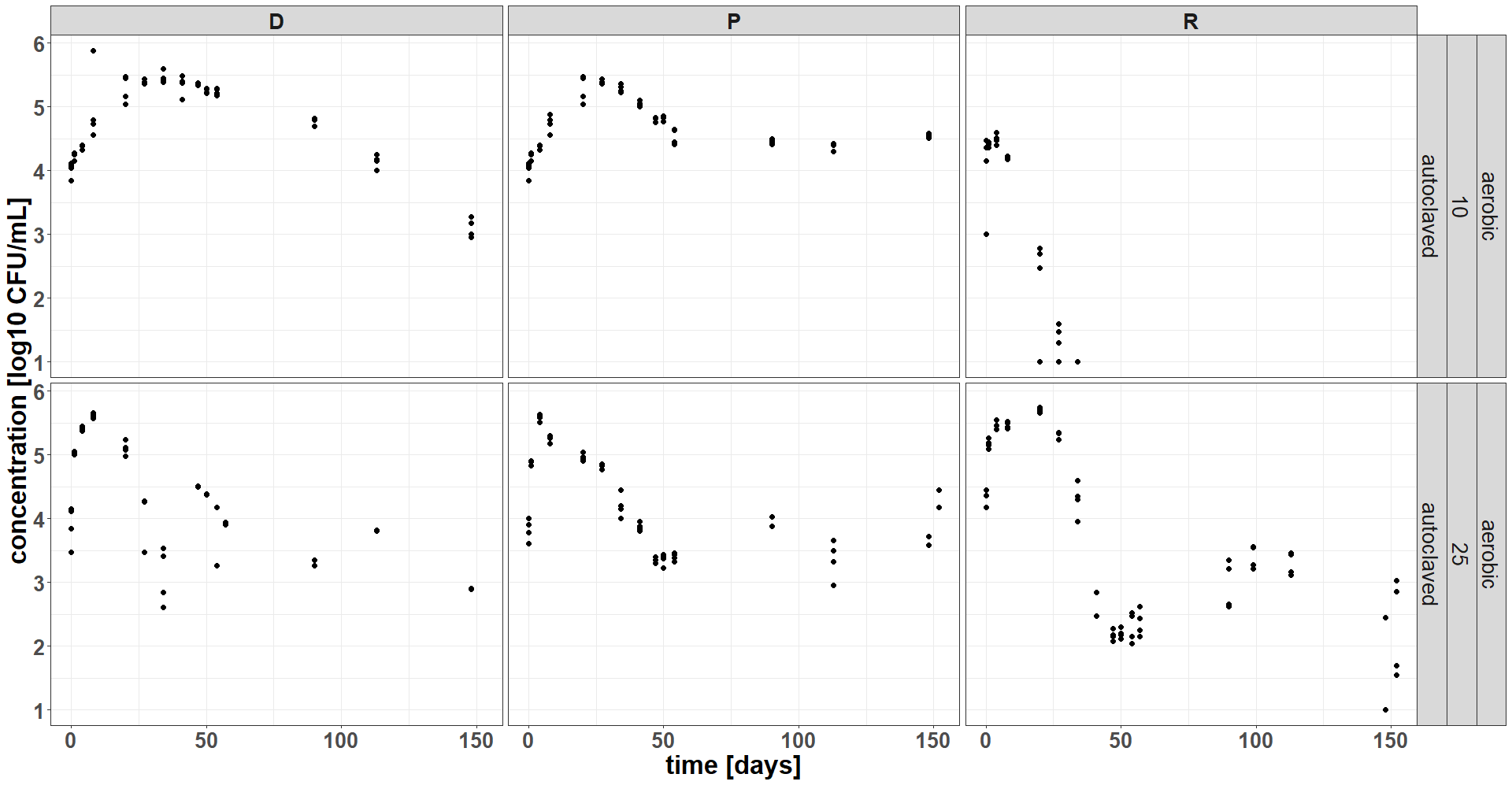

Supplement: S1 Fig — Die-off of Dickeya solani (D), Pectobacterium carotovorum sp. carotovorum (P), and Ralstonia solanacearum (R) in microcosms in autoclaved TDW at two temperatures (10°C, first row, and 25°C, second row), shown as log10 [CFU/mL] vs. time [days]. Points represent the plate counts in duplicate of two microcosms per treatment. (TIFF) [file pone.0250338.s001.tiff]
