# Supplementary material for: Die-off of plant pathogenic bacteria in tile drainage and anoxic water from a managed aquifer recharge site
Source: PLoS One. 2021 May 5;16(5):e0250338. doi: 10.1371/journal.pone.0250338 (PMC8099070; doi:10.1371/journal.pone.0250338)
Supplement: S1 Table — (DOCX) [file pone.0250338.s002.docx]

S1 Table. Enumeration of culturable bacteria in natural waters from a managed aquifer recharge system.

| 1. **Oxic microcosms in tile drainage water** | | | | | | | | | | | |
| --- | --- | --- | --- | --- | --- | --- | --- | --- | --- | --- | --- |
| 1. Oxic tile drainage water at 10 °C | | | | | 1. Oxic tile drainage water at 25 °C | | | | | | |
|  | days after inoculation* | microcosm | medium | CFU/mL |  | | | days after inoculation | microcosm | medium | CFU/mL |
| Control | 0 |  | TSA | 1.00 x10^2^ | Control | | | 0 |  | TSA | 2.00 x10^2^ |
|  | 16 |  | TSA | 9.56 x10^3^ |  |  |  | 16 |  | TSA | 2.32 x10^3^ |
|  |  |  | R2A | 9.80 x10^3^ |  |  |  |  |  | R2A | 1.00 x10^4^ |
|  | | | | | | | | | | | |
| Total culturable bacteria were also assessed at the end of pathogen die‑off period in microcosms of *Dickeya solani* and *Pectobacterium*  *carotovorum* sp. *carotovorum* (the pathogens were no more detectable by plating). The pathogen concentration of *Ralstonia solanacearum* was still 10^2^ CFU/mL by plating when this assessment was done. | | | | | | | | | | | |
|  | | | | | | | | | | | |
| *Ralstonia solanacearum* | 12 | 1 | TSA | 1.12 x10^4^ | *Ralstonia solanacearum* | | | 12 | 1 | TSA | 2.00 x10^3^ |
|  |  |  | R2A | 1.06 x10^4^ |  |  |  |  |  | R2A | 2.40 x10^3^ |
|  |  | 2 | TSA | 7.12 x10^3^ |  |  |  |  | 2 | TSA | 1.74 x10^3^ |
|  |  |  | R2A | 6.80 x10^3^ |  |  |  |  |  | R2A | 3.00 x10^3^ |
| *Dickeya solani* | 16 | 1 | TSA | 4.44 x10^3^ | *Dickeya solani* | | | 16 | 1 | TSA | 6.20 x10^3^ |
|  |  |  | R2A | 3.50 x10^3^ |  |  |  |  |  | R2A | 1.32 x10^4^ |
|  |  | 2 | TSA | 1.64 x10^3^ |  |  |  |  | 2 | TSA | 1.48 x10^3^ |
|  |  |  | R2A | 1.56 x10^3^ |  |  |  |  |  | R2A | 4.92 x10^3^ |
| *Pectobacterium*  *carotovorum* sp. *carotovorum* | 16 | 1 | TSA | 1.98 x10^3^ | *Pectobacterium*  *carotovorum* sp. *carotovorum* | | | 16 | 1 | TSA | 5.92 x10^3^ |
|  |  |  | R2A | 2.42 x10^3^ |  |  |  |  |  | R2A | 4.48 x10^3^ |
|  |  | 2 | TSA | 2.38 x10^3^ |  |  |  |  | 2 | TSA | 2.46 x10^3^ |
|  |  |  | R2A | 2.92 x10^3^ |  |  |  |  |  | R2A | - 1. x10^3^ |
|  | | | | | | | |  |  |  |  |
| 1. **Anoxic microcosm in aquifer water at 10 °C** | | | | | | | |  |  |  |  |
|  | days after inoculation* |  | medium | CFU/mL | | | | |  |  |  |
|  |  |  |  | plate 1 | | plate 2 | average | |  |  |  |
| Control  (no NO_3_) | 3 |  | TSA | 4.24 x10^3^ | | 3.16 x10^3^ | 3.70 x10^3^ | |  |  |  |
|  |  |  | R2A | 7.56 x10^3^ | | 4.28 x10^3^ | 5.92 x10^3^ | |  |  |  |
| Control  (NO_3_ added) | 3 |  | TSA | 3.28 x10^3^ | | 3.12 x10^3^ | 3.20 x10^3^ | |  |  |  |
|  |  |  | R2A | 5.08 x10^3^ | | 3.64 x10^3^ | 4.36 x10^3^ | |  |  |  |
| *Controls are non-inoculated water microcosm and the time indication refers to the start of the experiment when pathogen microcosms were inoculated. The total duration of the die‑off experiments can be followed in Figure 3. | | | | | | | | |  | |  |
