# Supplementary material for: Die-off of plant pathogenic bacteria in tile drainage and anoxic water from a managed aquifer recharge site
Source: PLoS One. 2021 May 5;16(5):e0250338. doi: 10.1371/journal.pone.0250338 (PMC8099070; doi:10.1371/journal.pone.0250338)
Supplement: S2 Table — (DOCX) [file pone.0250338.s003.docx]

S2 Table. Akaike information criterion (AIC) values; the minimum value among the three models is with a colored background and depicts the model with the best fit.

| **dataset** | |  | |  |  | | **Model type** | | |
| --- | --- | --- | --- | --- | --- | --- | --- | --- | --- |
|  |  | **[ᵒC]** | **treatment** | | | **redox** | **Weibull + tail** | **Weibull** | **Log-linear** |
| ***Ralstonia solanacearum*** | R1 | 10 | natural | | | oxic | 53 | 64 | 62 |
|  | R2 | 25 | natural | | | oxic | 109 | 108 | 118 |
|  | R3 | 10 | 0.22 µm filtered | | | oxic | - | 49 | 62 |
|  | R4 | 25 | 0.22 µm filtered | | | oxic | 121 | 145 | 157 |
|  | R5 | 10 | natural | | | anoxic | 181 | 224 | 223 |
|  | R6 | 10 | natural + NO_3_ | | | anoxic | 188 | 203 | 204 |
|  |  |  |  | | |  |  |  |  |
| ***Dickeya solani*** | D1 | 10 | natural | | | oxic | 48 | 53 | 54 |
|  | D2 | 25 | natural | | | oxic | - | 31 | 33 |
|  | D3 | 10 | 0.22 µm filtered | | | oxic | 69 | 79 | 128 |
|  | D4 | 25 | 0.22 µm filtered | | | oxic | - | 37 | 37 |
|  | D5 | 10 | natural | | | anoxic | - | 168 | 199 |
|  | D6 | 10 | natural + NO_3_ | | | anoxic | 111 | 111 | 133 |
|  |  |  |  | | |  |  |  |  |
| ***Pectobacterium carotovorum sp. carotovorum*** | P1 | 10 | natural | | | oxic | - | 32 | 54 |
|  | P2 | 25 | natural | | | oxic | - | 11 | 36 |
|  | P3 | 10 | 0.22 µm filtered | | | oxic | 73 | 85 | 110 |
|  | P4 | 25 | 0.22 µm filtered | | | oxic | - | 46 | 51 |
|  | P5 | 10 | natural | | | anoxic | 144 | 148 | 157 |
