# Supplementary material for: Die-off of plant pathogenic bacteria in tile drainage and anoxic water from a managed aquifer recharge site
Source: PLoS One. 2021 May 5;16(5):e0250338. doi: 10.1371/journal.pone.0250338 (PMC8099070; doi:10.1371/journal.pone.0250338)
Supplement: S1 File — (DOCX) [file pone.0250338.s004.docx]

**S1 File. R script for non-linear modeling**

Used in R studio Version 1.2.5033

Non-linear modelling

#ANALYSIS OF DIE-OFF OF PLANT PATHOGENIC BACTERIA

**# DATA**

# to read data from a spreadsheet

library( openxlsx )

# to manipulate data

library( dplyr )

#library(investr)

# set working directory (change this on your own computer)

setwd("C:/Data/R")

# read data aerobic experiment

#The spreadsheet looked like this (row 1, row 2 for explanation):

#

| day | O2 | water | bac | oc | flask | plate | c |
| --- | --- | --- | --- | --- | --- | --- | --- |
| days post inoculation | - aerobic  - anaerobic | Water type  - natural  - filtered  - autoclaved | bacterium in experiment  - R  - D  - P | temperature  - 10  - 25 | Per experiment duplicate flasks  - A (flask 1)  - B (flask 2) | All samples were plated in duplicates. Per flask and time point = 2 plate results  - a (result plate 1)  - b (result plate 2) | Concentration counted in [CFU/mL] |

data1 <- read.xlsx("data1", sheet=1, startRow = 1, check.names=T )

# include the natural log of c and make bac, flask and plate factors for testing; use “logc = log**10**( c ) )” to get graphs in log10

data1 <- data1 %>%

mutate(

o2 = as.factor("aerobic"),

water=as.factor(water),

bac=as.factor(bac),

flask=as.factor( flask ),

plate= as.factor(plate),

logc = log( c ) )%>%

select(day, o2, oc, water, bac, flask, plate, logc)

# read data anaerobic experiments

data2 <- read.xlsx("data2", sheet=2, startRow = 1, check.names=T )

# include the natural log of c and make bac, flask and plate factors for testing

data2 <- data2 %>%

mutate(

o2 = as.factor("anaerobic"),

water=as.factor(water),

bac=as.factor(bac),

flask=as.factor( flask ),

plate= as.factor(plate),

oc= as.numeric(10),

logc = log( c ) ) %>%

select(day, o2, oc, water, bac, flask, plate, logc)

# make one data set and remove data1 and data2

data <- rbind(data1, data2)

rm(data1)

rm(data2)

**# for plotting**

#the best fitting model (after model selection) will be plotted with the following function that includes the prediction interval, visualization of the detection limit and axis labels

library(investr)

# Function to plot the selected model

#A: y-axis in log scale

preddieoff <- function(myfit) {

plotFit(myfit,interval="prediction", pch=16,col.pred=c("#CCFFFF"),shade=T,

cex.axis=1.5, cex.lab=1.2, ylab= " ", xlab=" ") #expression('log' [10]*' [CFU/mL]'))

abline(h=log10(10), col="purple", lty=3)

}

#B: y-axis in log10 scale (but initial values of concentrations need also to be in log10 form)

preddieoff <- function(myfit) {

plotFit(myfit,interval="prediction", pch=16,col.pred=c("#CCFFFF"),shade=T, ylim=c(0,5),

cex.axis=1.5, cex.lab=1.2, ylab= " ", xlab=" ") #expression('log' [10]*' [CFU/mL]'))

abline(h=log10(10), col="purple", lty=3)

}

**# MODEL selection – which one fits best?**

**# Define model formulas**

#Weibull plus tail model

# determine the formulas for each model

WT <- as.formula(logc ~ log((exp(logc0)-exp(logcres))*exp(-(a*day)^b)+exp(logcres)))

#Weibull model

W <- as.formula(logc~logc0-(a*day)^b)

#Log-linear model

L <- as.formula(logc~logc0-(a*day))

#Apply models to the dataset, which one is best fit?

#select the dataset you want to analyse

seldata <- data %>% filter(bac=="R", o2=="aerobic", oc==10, water=="natural")

par(mfrow=c(3,2)) #all three models will be displayed in the same window

#preview function: selected model needs starting values, the function displays a first fit and can be used to adapt the starting values (if starting values are poor, the fit function nls function to model will NOT work)

**#____________________________________________________**

**# mod1: Weibull plus tail**

preview(WT, data = seldata, start = list(logc0=10, logcres=4, a=0.2, b=4))

# fit the model

fit1 <- nls(WT, data = seldata, start = list(logc0=10, logcres=4, a=0.2, b=4))

#show the fitted model and data

plotfit(fit1, smooth = TRUE)

**#____________________________________________________**

**# mod2: Weibull**

mod <- as.formula(logc~logc0-(a*day)^b)

# show the data and preview of the model prediction for some good guesses of parameter values (manual fitting)

preview(W, data = seldata, start = list(logc0=10, a=0.15, b=4))

# fit the model

fit2 <- nls(W, data = seldata, start = list(logc0=10, a=0.15, b=4))

#show the fitted model and data

plotfit(fit2, smooth = TRUE)

**#____________________________________________________**

**# mod3: loglinear**

mod <- as.formula(logc~logc0-a*day)

# show the data and preview of the model prediction for some good guesses of parameter values (manual fitting)

preview(L, data = seldata, start = list(logc0=10, a=0.2))

# fit the model

fit3 <- nls(L, data = seldata, start = list(logc0=10, a=0.2))

#show the fitted model and data

plotfit(fit3, smooth = TRUE)

**#____________________________________________________**

**# model comparison**

# lowest AIC is best model

AIC(fit1,fit2,fit3)

# anova

a<-anova(fit1,fit2, fit3)

a

# selecting the model that fitted best (see AIC and anova)

bestfit <- fit1

overview(bestfit) #shows values of parameter estimates

#makes a data frame out of the data and export to excel

mytidybestfit <- tidy(bestfit)

# fitting results and statistics

res <- nlsResiduals(bestfit)

# evaluation of residuals

plot(res)

# test for normality

test.nlsResiduals(res)

# prediction, plot the final graph

preddieoff(bestfit)

title( main = "R, aerobic, 10 oC, natural water")

**# Do the tested conditions have a significant influence on the die-off of the bacteria?**

**# influence of nitrate on the die-off of R. solanacearum in anoxic natural microcosms at 10C**

library(nlme)

watermod <- nlsList(

logc ~ log((exp(logc0)-exp(logcres))*exp(-(a*day)^b)+exp(logcres))| water ,

data = data %>% filter(bac=="R", o2=="anaerobic", oc==10),

start = list(logc0=8, logcres=3, a=0.06, b=2)

)

tidy(watermod)

mod <- nls(

logc ~ log((exp(logc0)-exp(logcres))*exp(-(a*day)^b)+exp(logcres)),

data = data %>% filter(bac=="R", o2=="anaerobic", oc==10),

start = list(logc0=8, logcres=3, a=0.06, b=2)

)

tidy(mod)

library(nlshelper)

tidygroup<- tidy(anova_nlslist(watermod,mod))
