# Supplementary material for: Die-off of plant pathogenic bacteria in tile drainage and anoxic water from a managed aquifer recharge site
Source: PLoS One. 2021 May 5;16(5):e0250338. doi: 10.1371/journal.pone.0250338 (PMC8099070; doi:10.1371/journal.pone.0250338)
Supplement: S2 File — (PDF) [file pone.0250338.s005.pdf]

S2_File. Experimental data to model die-off in different water types under oxic and anoxic conditions

options for anaerobic setup							
parameter:	day	water	bac	oc	flask	plate	c
		nitrate	R	=temp, but all experiments	A	a	
		nonit	D	at 10	B	b	
			P				
**aerobic setup**							
parameter:	day	water	bac	oc	flask	plate	c
		natural	R	warm	A	a	
		filtered	D	cold	B	b	
		autoclaved	P				
			R	Ralstonia solanacearum			
			D	Dickeya solani			
			P	Pectobacterium carotovorum sp. carotovorum.			

Die-off of plant pathogenic bacteria in anoxic aquifer water

day	water	bac	flask	plate	c
0	nonit	P	A	a	4000
1	nonit	P	A	a	2700
2	nonit	P	A	a	3300
3	nonit	P	A	a	4280
4	nonit	P	A	a	3800
5	nonit	P	A	a	3400
6	nonit	P	A	a	3860
7	nonit	P	A	a	5360
8	nonit	P	A	a	1770
9	nonit	P	A	a	2990
23	nonit	P	A	a	930
25	nonit	P	A	a	220
27	nonit	P	A	a	270
30	nonit	P	A	a	240
34	nonit	P	A	a	30
36	nonit	P	A	a	140
38	nonit	P	A	a	30
41	nonit	P	A	a	15
43	nonit	P	A	a	20
45	nonit	P	A	a	40
0	nonit	P	A	b	3000
1	nonit	P	A	b	1400
2	nonit	P	A	b	1500
3	nonit	P	A	b	4320
4	nonit	P	A	b	7060
5	nonit	P	A	b	1540
6	nonit	P	A	b	3080
7	nonit	P	A	b	3720
8	nonit	P	A	b	2000
9	nonit	P	A	b	1740
23	nonit	P	A	b	1500
25	nonit	P	A	b	290
27	nonit	P	A	b	200
30	nonit	P	A	b	80
34	nonit	P	A	b	50
36	nonit	P	A	b	70
38	nonit	P	A	b	30
41	nonit	P	A	b	75
43	nonit	P	A	b	10
45	nonit	P	A	b	55
0	nonit	P	B	a	2000
1	nonit	P	B	a	4300
2	nonit	P	B	a	1700
3	nonit	P	B	a	6460
4	nonit	P	B	a	3320
5	nonit	P	B	a	1760

6	nonit	P	B	a	2330
7	nonit	P	B	a	1830
8	nonit	P	B	a	1780
9	nonit	P	B	a	2860
23	nonit	P	B	a	340
25	nonit	P	B	a	270
27	nonit	P	B	a	150
30	nonit	P	B	a	170
34	nonit	P	B	a	70
36	nonit	P	B	a	110
38	nonit	P	B	a	60
41	nonit	P	B	a	15
43	nonit	P	B	a	10
45	nonit	P	B	a	10
0	nonit	P	B	b	9000
1	nonit	P	B	b	2600
2	nonit	P	B	b	4100
3	nonit	P	B	b	6360
4	nonit	P	B	b	4780
5	nonit	P	B	b	1750
6	nonit	P	B	b	3230
7	nonit	P	B	b	2280
8	nonit	P	B	b	2260
9	nonit	P	B	b	2140
23	nonit	P	B	b	1420
25	nonit	P	B	b	170
27	nonit	P	B	b	140
30	nonit	P	B	b	190
34	nonit	P	B	b	20
36	nonit	P	B	b	100
38	nonit	P	B	b	10
41	nonit	P	B	b	40
43	nonit	P	B	b	10
0	nonit	D	A	a	12000
1	nonit	D	A	a	2800
2	nonit	D	A	a	2800
3	nonit	D	A	a	8420
4	nonit	D	A	a	7080
5	nonit	D	A	a	2120
7	nonit	D	A	a	8900
8	nonit	D	A	a	8100
9	nonit	D	A	a	7200
23	nonit	D	A	a	3390
30	nonit	D	A	a	850
34	nonit	D	A	a	400
36	nonit	D	A	a	620
38	nonit	D	A	a	520

41	nonit	D	A	a	670
43	nonit	D	A	a	340
45	nonit	D	A	a	450
48	nonit	D	A	a	40
0	nonit	D	A	b	7000
1	nonit	D	A	b	3000
2	nonit	D	A	b	6100
3	nonit	D	A	b	7000
4	nonit	D	A	b	9060
5	nonit	D	A	b	4320
7	nonit	D	A	b	5680
8	nonit	D	A	b	8700
9	nonit	D	A	b	8100
23	nonit	D	A	b	2500
30	nonit	D	A	b	890
34	nonit	D	A	b	410
36	nonit	D	A	b	790
38	nonit	D	A	b	300
41	nonit	D	A	b	720
43	nonit	D	A	b	560
45	nonit	D	A	b	380
48	nonit	D	A	b	170
0	nonit	D	B	a	8000
1	nonit	D	B	a	4500
2	nonit	D	B	a	9700
3	nonit	D	B	a	7920
4	nonit	D	B	a	4760
5	nonit	D	B	a	4040
7	nonit	D	B	a	5060
8	nonit	D	B	a	9500
9	nonit	D	B	a	4700
23	nonit	D	B	a	2360
30	nonit	D	B	a	520
34	nonit	D	B	a	460
36	nonit	D	B	a	200
38	nonit	D	B	a	920
41	nonit	D	B	a	210
43	nonit	D	B	a	70
45	nonit	D	B	a	100
48	nonit	D	B	a	30
0	nonit	D	B	b	7000
1	nonit	D	B	b	5800
2	nonit	D	B	b	8500
3	nonit	D	B	b	5700
4	nonit	D	B	b	4180
5	nonit	D	B	b	3350
7	nonit	D	B	b	5180

8	nonit	D	B	b	8200
9	nonit	D	B	b	8700
23	nonit	D	B	b	1860
30	nonit	D	B	b	970
34	nonit	D	B	b	400
36	nonit	D	B	b	490
38	nonit	D	B	b	590
41	nonit	D	B	b	410
43	nonit	D	B	b	80
45	nonit	D	B	b	140
48	nonit	D	B	b	50
0	nonit	R	A	a	42000
1	nonit	R	A	a	19300
2	nonit	R	A	a	25700
3	nonit	R	A	a	11240
4	nonit	R	A	a	8720
5	nonit	R	A	a	16120
6	nonit	R	A	a	15800
7	nonit	R	A	a	9280
8	nonit	R	A	a	10640
8	nonit	R	A	a	25100
9	nonit	R	A	a	50600
11	nonit	R	A	a	19100
23	nonit	R	A	a	320
25	nonit	R	A	a	110
27	nonit	R	A	a	140
30	nonit	R	A	a	30
34	nonit	R	A	a	200
36	nonit	R	A	a	60
38	nonit	R	A	a	45
41	nonit	R	A	a	10
43	nonit	R	A	a	35
45	nonit	R	A	a	25
0	nonit	R	A	b	45000
2	nonit	R	A	b	22100
3	nonit	R	A	b	7220
4	nonit	R	A	b	7040
5	nonit	R	A	b	19480
6	nonit	R	A	b	9480
7	nonit	R	A	b	5400
8	nonit	R	A	b	31300
9	nonit	R	A	b	50900
11	nonit	R	A	b	21400
23	nonit	R	A	b	170
25	nonit	R	A	b	300
30	nonit	R	A	b	60
34	nonit	R	A	b	70

36	nonit	R	A	b	65
38	nonit	R	A	b	40
41	nonit	R	A	b	40
43	nonit	R	A	b	10
45	nonit	R	A	b	15
0	nonit	R	B	a	17000
1	nonit	R	B	a	21300
2	nonit	R	B	a	23700
3	nonit	R	B	a	8140
4	nonit	R	B	a	16140
5	nonit	R	B	a	7400
6	nonit	R	B	a	6440
7	nonit	R	B	a	2860
8	nonit	R	B	a	7960
8	nonit	R	B	a	30100
9	nonit	R	B	a	44500
11	nonit	R	B	a	25200
23	nonit	R	B	a	2520
25	nonit	R	B	a	80
27	nonit	R	B	a	10
30	nonit	R	B	a	10
36	nonit	R	B	a	10
0	nonit	R	B	b	18000
2	nonit	R	B	b	20100
3	nonit	R	B	b	8500
4	nonit	R	B	b	14400
5	nonit	R	B	b	13880
6	nonit	R	B	b	12920
7	nonit	R	B	b	4000
8	nonit	R	B	b	26700
9	nonit	R	B	b	43600
11	nonit	R	B	b	20700
23	nonit	R	B	b	2070
25	nonit	R	B	b	170
27	nonit	R	B	b	90
30	nonit	R	B	b	20
36	nonit	R	B	b	100
0	nitrate	D	A	a	7000
1	nitrate	D	A	a	8700
2	nitrate	D	A	a	7300
3	nitrate	D	A	a	11140
4	nitrate	D	A	a	8980
5	nitrate	D	A	a	7180
7	nitrate	D	A	a	9520
8	nitrate	D	A	a	9700
9	nitrate	D	A	a	14100
23	nitrate	D	A	a	5520

23	nitrate	D	A	a	5400
30	nitrate	D	A	a	1750
34	nitrate	D	A	a	580
36	nitrate	D	A	a	1850
38	nitrate	D	A	a	1580
41	nitrate	D	A	a	490
43	nitrate	D	A	a	200
45	nitrate	D	A	a	60
48	nitrate	D	A	a	30
0	nonit	D	A	b	8000
1	nonit	D	A	b	8800
2	nonit	D	A	b	7800
3	nonit	D	A	b	12140
4	nonit	D	A	b	10780
5	nonit	D	A	b	9480
7	nonit	D	A	b	6080
8	nonit	D	A	b	11300
23	nonit	D	A	b	12600
23	nonit	D	A	b	2600
30	nonit	D	A	b	2560
34	nonit	D	A	b	510
36	nonit	D	A	b	2640
38	nonit	D	A	b	280
41	nonit	D	A	b	520
43	nonit	D	A	b	130
45	nonit	D	A	b	60
48	nonit	D	A	b	20
0	nitrate	D	B	a	11000
1	nitrate	D	B	a	5000
2	nitrate	D	B	a	9100
3	nitrate	D	B	a	8920
4	nitrate	D	B	a	8580
5	nitrate	D	B	a	11000
7	nitrate	D	B	a	10960
8	nitrate	D	B	a	9300
9	nitrate	D	B	a	11800
23	nitrate	D	B	a	4790
23	nitrate	D	B	a	6600
30	nitrate	D	B	a	730
34	nitrate	D	B	a	390
36	nitrate	D	B	a	440
38	nitrate	D	B	a	240
0	nitrate	D	B	b	10000
1	nitrate	D	B	b	6700
2	nitrate	D	B	b	7200
3	nitrate	D	B	b	10500
4	nitrate	D	B	b	9000

5	nitrate	D	B	b	9080
6	nitrate	D	B	b	4960
7	nitrate	D	B	b	12800
8	nitrate	D	B	b	12100
23	nitrate	D	B	b	4120
30	nitrate	D	B	b	760
34	nitrate	D	B	b	370
36	nitrate	D	B	b	200
38	nitrate	D	B	b	40
41	nitrate	D	B	b	10
0	nitrate	R	A	a	26000
1	nitrate	R	A	a	26700
2	nitrate	R	A	a	30900
3	nitrate	R	A	a	10960
4	nitrate	R	A	a	8720
5	nitrate	R	A	a	15160
6	nitrate	R	A	a	14840
7	nitrate	R	A	a	11600
8	nitrate	R	A	a	8320
8	nitrate	R	A	a	29800
9	nitrate	R	A	a	44300
11	nitrate	R	A	a	34500
23	nitrate	R	A	a	290
25	nitrate	R	A	a	190
27	nitrate	R	A	a	260
30	nitrate	R	A	a	30
34	nitrate	R	A	a	60
36	nitrate	R	A	a	25
38	nitrate	R	A	a	80
41	nitrate	R	A	a	10
48	nitrate	R	A	a	5
0	nitrate	R	A	b	37000
2	nitrate	R	A	b	31700
3	nitrate	R	A	b	22720
4	nitrate	R	A	b	27120
5	nitrate	R	A	b	13800
6	nitrate	R	A	b	8080
7	nitrate	R	A	b	9360
8	nitrate	R	A	b	31100
9	nitrate	R	A	b	41800
11	nitrate	R	A	b	33900
23	nitrate	R	A	b	270
25	nitrate	R	A	b	270
27	nitrate	R	A	b	50
30	nitrate	R	A	b	30
34	nitrate	R	A	b	90
36	nitrate	R	A	b	80

38	nitrate	R	A	b	70
41	nitrate	R	A	b	5
43	nitrate	R	A	b	35
45	nitrate	R	A	b	5
0	nitrate	R	B	a	21000
1	nitrate	R	B	a	17000
2	nitrate	R	B	a	21200
3	nitrate	R	B	a	9740
4	nitrate	R	B	a	21600
5	nitrate	R	B	a	10000
6	nitrate	R	B	a	9960
7	nitrate	R	B	a	5080
8	nitrate	R	B	a	15320
8	nitrate	R	B	a	26900
9	nitrate	R	B	a	44500
11	nitrate	R	B	a	29000
23	nitrate	R	B	a	440
25	nitrate	R	B	a	110
27	nitrate	R	B	a	120
34	nitrate	R	B	a	30
36	nitrate	R	B	a	40
38	nitrate	R	B	a	35
43	nitrate	R	B	a	5
0	nitrate	R	B	b	27000
2	nitrate	R	B	b	25900
3	nitrate	R	B	b	14820
4	nitrate	R	B	b	8200
5	nitrate	R	B	b	12800
6	nitrate	R	B	b	14160
7	nitrate	R	B	b	4240
8	nitrate	R	B	b	25400
9	nitrate	R	B	b	44500
11	nitrate	R	B	b	21700
23	nitrate	R	B	b	170
25	nitrate	R	B	b	170
27	nitrate	R	B	b	60
34	nitrate	R	B	b	40
36	nitrate	R	B	b	40
38	nitrate	R	B	b	10
41	nitrate	R	B	b	10
43	nitrate	R	B	b	5

Die-off of plant pathogenic bacteria in oxic tile drainage water

day	water	bac	oc	flask	plate	c
0	natural	R	cold	A	a	10700
2	natural	R	cold	A	a	16800
4	natural	R	cold	A	a	9900
10	natural	R	cold	A	a	160
0	natural	R	cold	A	b	12300
2	natural	R	cold	A	b	17000
4	natural	R	cold	A	b	10000
10	natural	R	cold	A	b	70
0	natural	R	cold	B	a	14200
2	natural	R	cold	B	a	18400
4	natural	R	cold	B	a	10500
8	natural	R	cold	B	a	600
10	natural	R	cold	B	a	200
12	natural	R	cold	B	a	10
0	natural	R	cold	B	b	15400
2	natural	R	cold	B	b	19700
4	natural	R	cold	B	b	8900
8	natural	R	cold	B	b	300
10	natural	R	cold	B	b	130
12	natural	R	cold	B	b	90
0	natural	D	cold	A	a	13000
2	natural	D	cold	A	a	14400
6	natural	D	cold	A	a	7000
8	natural	D	cold	A	a	200
0	natural	D	cold	A	b	10000
2	natural	D	cold	A	b	14600
6	natural	D	cold	A	b	4000
8	natural	D	cold	A	b	700
0	natural	D	cold	B	a	107000
2	natural	D	cold	B	a	16100
6	natural	D	cold	B	a	6000
8	natural	D	cold	B	a	300
12	natural	D	cold	B	a	30
0	natural	D	cold	B	b	143000
2	natural	D	cold	B	b	17100
6	natural	D	cold	B	b	8000
8	natural	D	cold	B	b	300
12	natural	D	cold	B	b	80
0	natural	P	cold	A	a	6700
1	natural	P	cold	A	a	7200
2	natural	P	cold	A	a	14400
6	natural	P	cold	A	a	9000
8	natural	P	cold	A	a	200
0	natural	P	cold	A	b	6800
1	natural	P	cold	A	b	8520

2	natural	P	cold	A	b	13700
6	natural	P	cold	A	b	7000
8	natural	P	cold	A	b	800
0	natural	P	cold	B	a	5800
1	natural	P	cold	B	a	5700
2	natural	P	cold	B	a	15300
6	natural	P	cold	B	a	3000
8	natural	P	cold	B	a	400
0	natural	P	cold	B	b	8600
1	natural	P	cold	B	b	6580
2	natural	P	cold	B	b	16200
6	natural	P	cold	B	b	3000
8	natural	P	cold	B	b	600
0	natural	R	warm	A	a	13200
2	natural	R	warm	A	a	24300
3	natural	R	warm	A	a	3400
4	natural	R	warm	A	a	1100
8	natural	R	warm	A	a	470
10	natural	R	warm	A	a	210
12	natural	R	warm	A	a	90
15	natural	R	warm	A	a	300
19	natural	R	warm	A	a	50
22	natural	R	warm	A	a	30
0	natural	R	warm	A	b	15200
2	natural	R	warm	A	b	28300
3	natural	R	warm	A	b	3200
4	natural	R	warm	A	b	1200
8	natural	R	warm	A	b	580
10	natural	R	warm	A	b	230
12	natural	R	warm	A	b	50
15	natural	R	warm	A	b	320
19	natural	R	warm	A	b	70
22	natural	R	warm	A	b	10
25	natural	R	warm	A	b	10
0	natural	R	warm	B	a	10800
2	natural	R	warm	B	a	21400
3	natural	R	warm	B	a	2100
4	natural	R	warm	B	a	700
8	natural	R	warm	B	a	250
10	natural	R	warm	B	a	70
15	natural	R	warm	B	a	110
19	natural	R	warm	B	a	70
22	natural	R	warm	B	a	50
0	natural	R	warm	B	b	16200
2	natural	R	warm	B	b	18300
3	natural	R	warm	B	b	2000
4	natural	R	warm	B	b	400

8	natural	R	warm	B	b	400
10	natural	R	warm	B	b	100
12	natural	R	warm	B	b	110
15	natural	R	warm	B	b	180
19	natural	R	warm	B	b	70
22	natural	R	warm	B	b	20
0	natural	D	warm	A	a	18000
2	natural	D	warm	A	a	300
4	natural	D	warm	A	a	100
0	natural	D	warm	A	b	20800
2	natural	D	warm	A	b	500
4	natural	D	warm	A	b	200
0	natural	D	warm	B	a	15000
2	natural	D	warm	B	a	2200
4	natural	D	warm	B	a	300
0	natural	D	warm	B	b	16000
2	natural	D	warm	B	b	1700
4	natural	D	warm	B	b	700
0	natural	P	warm	A	a	7500
1	natural	P	warm	A	a	10060
2	natural	P	warm	A	a	4500
4	natural	P	warm	A	a	400
0	natural	P	warm	A	b	6900
1	natural	P	warm	A	b	12380
2	natural	P	warm	A	b	3800
4	natural	P	warm	A	b	400
0	natural	P	warm	B	a	6400
1	natural	P	warm	B	a	8840
2	natural	P	warm	B	a	5600
4	natural	P	warm	B	a	200
0	natural	P	warm	B	b	7300
1	natural	P	warm	B	b	13220
2	natural	P	warm	B	b	5200
4	natural	P	warm	B	b	300
0	filtered	R	cold	A	a	11000
4	filtered	R	cold	A	a	8500
7	filtered	R	cold	A	a	21500
14	filtered	R	cold	A	a	1000
19	filtered	R	cold	A	a	90
25	filtered	R	cold	A	a	3
0	filtered	R	cold	A	b	14000
4	filtered	R	cold	A	b	8700
7	filtered	R	cold	A	b	7100
14	filtered	R	cold	A	b	1300
19	filtered	R	cold	A	b	80
0	filtered	R	cold	B	a	19000
4	filtered	R	cold	B	a	1170

7	filtered	R	cold	B	a	8700
14	filtered	R	cold	B	a	800
19	filtered	R	cold	B	a	530
0	filtered	R	cold	B	b	9000
4	filtered	R	cold	B	b	10800
7	filtered	R	cold	B	b	9700
14	filtered	R	cold	B	b	700
0	filtered	D	cold	A	a	6000
3	filtered	D	cold	A	a	8000
14	filtered	D	cold	A	a	11200
17	filtered	D	cold	A	a	10300
27	filtered	D	cold	A	a	8900
30	filtered	D	cold	A	a	5000
41	filtered	D	cold	A	a	100
43	filtered	D	cold	A	a	280
45	filtered	D	cold	A	a	110
50	filtered	D	cold	A	a	10
0	filtered	D	cold	A	b	8000
3	filtered	D	cold	A	b	19000
14	filtered	D	cold	A	b	11800
17	filtered	D	cold	A	b	10300
27	filtered	D	cold	A	b	6400
30	filtered	D	cold	A	b	5100
43	filtered	D	cold	A	b	270
45	filtered	D	cold	A	b	60
50	filtered	D	cold	A	b	30
0	filtered	D	cold	B	a	8000
3	filtered	D	cold	B	a	13000
14	filtered	D	cold	B	a	8600
17	filtered	D	cold	B	a	10000
27	filtered	D	cold	B	a	6500
30	filtered	D	cold	B	a	4300
41	filtered	D	cold	B	a	800
43	filtered	D	cold	B	a	40
45	filtered	D	cold	B	a	10
0	filtered	D	cold	B	b	13000
3	filtered	D	cold	B	b	6000
14	filtered	D	cold	B	b	11000
17	filtered	D	cold	B	b	10200
27	filtered	D	cold	B	b	5400
30	filtered	D	cold	B	b	3500
41	filtered	D	cold	B	b	300
43	filtered	D	cold	B	b	120
45	filtered	D	cold	B	b	20
0	filtered	P	cold	A	a	6000
3	filtered	P	cold	A	a	11000

9	filtered	P	cold	A	a	6800
14	filtered	P	cold	A	a	4700
17	filtered	P	cold	A	a	2800
27	filtered	P	cold	A	a	1700
30	filtered	P	cold	A	a	950
41	filtered	P	cold	A	a	70
43	filtered	P	cold	A	a	10
45	filtered	P	cold	A	a	40
50	filtered	P	cold	A	a	20
0	filtered	P	cold	A	b	6000
3	filtered	P	cold	A	b	16000
9	filtered	P	cold	A	b	7800
14	filtered	P	cold	A	b	4800
17	filtered	P	cold	A	b	900
27	filtered	P	cold	A	b	1500
30	filtered	P	cold	A	b	890
41	filtered	P	cold	A	b	40
45	filtered	P	cold	A	b	100
50	filtered	P	cold	A	b	20
0	filtered	P	cold	B	a	7000
3	filtered	P	cold	B	a	11000
9	filtered	P	cold	B	a	6100
14	filtered	P	cold	B	a	4500
17	filtered	P	cold	B	a	4000
27	filtered	P	cold	B	a	1300
30	filtered	P	cold	B	a	1380
41	filtered	P	cold	B	a	70
43	filtered	P	cold	B	a	10
45	filtered	P	cold	B	a	40
0	filtered	P	cold	B	b	5000
3	filtered	P	cold	B	b	3000
9	filtered	P	cold	B	b	6800
14	filtered	P	cold	B	b	4300
17	filtered	P	cold	B	b	3800
27	filtered	P	cold	B	b	1600
30	filtered	P	cold	B	b	1230
41	filtered	P	cold	B	b	40
43	filtered	P	cold	B	b	20
45	filtered	P	cold	B	b	20
0	filtered	R	warm	A	a	21000
7	filtered	R	warm	A	a	8000
14	filtered	R	warm	A	a	500
25	filtered	R	warm	A	a	27
28	filtered	R	warm	A	a	39
32	filtered	R	warm	A	a	18
35	filtered	R	warm	A	a	21

39	filtered	R	warm A	a	9
42	filtered	R	warm A	a	18
46	filtered	R	warm A	a	45
52	filtered	R	warm A	a	3
0	filtered	R	warm A	b	14500
7	filtered	R	warm A	b	14000
14	filtered	R	warm A	b	800
19	filtered	R	warm A	b	10
25	filtered	R	warm A	b	84
28	filtered	R	warm A	b	12
32	filtered	R	warm A	b	39
35	filtered	R	warm A	b	27
39	filtered	R	warm A	b	12
42	filtered	R	warm A	b	30
46	filtered	R	warm A	b	96
52	filtered	R	warm A	b	15
0	filtered	R	warm B	a	16000
7	filtered	R	warm B	a	12000
14	filtered	R	warm B	a	700
25	filtered	R	warm B	a	39
32	filtered	R	warm B	a	30
35	filtered	R	warm B	a	3
39	filtered	R	warm B	a	15
42	filtered	R	warm B	a	21
46	filtered	R	warm B	a	24
52	filtered	R	warm B	a	3
60	filtered	R	warm B	a	3
0	filtered	R	warm B	b	14500
7	filtered	R	warm B	b	14000
14	filtered	R	warm B	b	400
19	filtered	R	warm B	b	110
28	filtered	R	warm B	b	27
32	filtered	R	warm B	b	21
35	filtered	R	warm B	b	6
39	filtered	R	warm B	b	18
42	filtered	R	warm B	b	21
46	filtered	R	warm B	b	30
52	filtered	R	warm B	b	3
0	filtered	D	warm A	a	9000
3	filtered	D	warm A	a	16000
9	filtered	D	warm A	a	100
14	filtered	D	warm A	a	100
0	filtered	D	warm A	b	15000
3	filtered	D	warm A	b	18000
14	filtered	D	warm A	b	120
0	filtered	D	warm B	a	11000
3	filtered	D	warm B	a	20000

14	filtered	D	warm	B	a	90
0	filtered	D	warm	B	b	11000
3	filtered	D	warm	B	b	9000
14	filtered	D	warm	B	b	70
0	filtered	P	warm	A	a	2000
3	filtered	P	warm	A	a	12000
9	filtered	P	warm	A	a	700
14	filtered	P	warm	A	a	30
0	filtered	P	warm	A	b	9000
3	filtered	P	warm	A	b	3000
9	filtered	P	warm	A	b	1000
14	filtered	P	warm	A	b	70
0	filtered	P	warm	B	a	4000
3	filtered	P	warm	B	a	14000
9	filtered	P	warm	B	a	1000
14	filtered	P	warm	B	a	50
17	filtered	P	warm	B	a	50
0	filtered	P	warm	B	b	6000
3	filtered	P	warm	B	b	15000
9	filtered	P	warm	B	b	1200
14	filtered	P	warm	B	b	60
17	filtered	P	warm	B	b	30
0	autoclaved	R	cold	A	a	23000
1	autoclaved	R	cold	A	a	25600
4	autoclaved	R	cold	A	a	25000
8	autoclaved	R	cold	A	a	15300
20	autoclaved	R	cold	A	a	500
27	autoclaved	R	cold	A	a	20
0	autoclaved	R	cold	A	b	30000
1	autoclaved	R	cold	A	b	27300
4	autoclaved	R	cold	A	b	39000
8	autoclaved	R	cold	A	b	16800
20	autoclaved	R	cold	A	b	300
27	autoclaved	R	cold	A	b	40
34	autoclaved	R	cold	A	b	10
0	autoclaved	R	cold	B	a	1000
1	autoclaved	R	cold	B	a	27700
4	autoclaved	R	cold	B	a	30000
8	autoclaved	R	cold	B	a	15600
20	autoclaved	R	cold	B	a	600
27	autoclaved	R	cold	B	a	30
34	autoclaved	R	cold	B	a	10
0	autoclaved	R	cold	B	b	14000
1	autoclaved	R	cold	B	b	23200
4	autoclaved	R	cold	B	b	32000
8	autoclaved	R	cold	B	b	15200

20	autoclaved	R	cold	B	b	10
27	autoclaved	R	cold	B	b	10
0	autoclaved	D	cold	A	a	13000
1	autoclaved	D	cold	A	a	14000
4	autoclaved	D	cold	A	a	20900
8	autoclaved	D	cold	A	a	62600
20	autoclaved	D	cold	A	a	280000
27	autoclaved	D	cold	A	a	270000
34	autoclaved	D	cold	A	a	266000
41	autoclaved	D	cold	A	a	236000
47	autoclaved	D	cold	A	a	226000
50	autoclaved	D	cold	A	a	189000
54	autoclaved	D	cold	A	a	186000
90	autoclaved	D	cold	A	a	65000
113	autoclaved	D	cold	A	a	18000
148	autoclaved	D	cold	A	a	1900
0	autoclaved	D	cold	A	b	12000
1	autoclaved	D	cold	A	b	18000
4	autoclaved	D	cold	A	b	25000
8	autoclaved	D	cold	A	b	53900
20	autoclaved	D	cold	A	b	145000
27	autoclaved	D	cold	A	b	245000
34	autoclaved	D	cold	A	b	244000
41	autoclaved	D	cold	A	b	129000
47	autoclaved	D	cold	A	b	218000
50	autoclaved	D	cold	A	b	163000
54	autoclaved	D	cold	A	b	151000
90	autoclaved	D	cold	A	b	66000
113	autoclaved	D	cold	A	b	10000
148	autoclaved	D	cold	A	b	1000
0	autoclaved	D	cold	B	a	11000
1	autoclaved	D	cold	B	a	18000
4	autoclaved	D	cold	B	a	25200
8	autoclaved	D	cold	B	a	752000
20	autoclaved	D	cold	B	a	299000
27	autoclaved	D	cold	B	a	275000
34	autoclaved	D	cold	B	a	282000
41	autoclaved	D	cold	B	a	251000
47	autoclaved	D	cold	B	a	233000
50	autoclaved	D	cold	B	a	191000
54	autoclaved	D	cold	B	a	165000
90	autoclaved	D	cold	B	a	62000
113	autoclaved	D	cold	B	a	14000
148	autoclaved	D	cold	B	a	900
0	autoclaved	D	cold	B	b	7000
1	autoclaved	D	cold	B	b	19000
4	autoclaved	D	cold	B	b	24300

8	autoclaved	D	cold	B	b	36500
20	autoclaved	D	cold	B	b	111000
27	autoclaved	D	cold	B	b	232000
34	autoclaved	D	cold	B	b	396000
41	autoclaved	D	cold	B	b	309000
47	autoclaved	D	cold	B	b	237000
50	autoclaved	D	cold	B	b	166000
54	autoclaved	D	cold	B	b	192000
90	autoclaved	D	cold	B	b	50000
113	autoclaved	D	cold	B	b	15000
148	autoclaved	D	cold	B	b	1500
0	autoclaved	P	cold	A	a	13000
1	autoclaved	P	cold	A	a	14000
4	autoclaved	P	cold	A	a	20900
8	autoclaved	P	cold	A	a	62600
20	autoclaved	P	cold	A	a	280000
27	autoclaved	P	cold	A	a	270000
34	autoclaved	P	cold	A	a	206000
41	autoclaved	P	cold	A	a	105000
47	autoclaved	P	cold	A	a	57000
50	autoclaved	P	cold	A	a	68200
54	autoclaved	P	cold	A	a	27800
90	autoclaved	P	cold	A	a	31300
113	autoclaved	P	cold	A	a	19900
148	autoclaved	P	cold	A	a	33500
0	autoclaved	P	cold	A	b	12000
1	autoclaved	P	cold	A	b	18000
4	autoclaved	P	cold	A	b	25000
8	autoclaved	P	cold	A	b	53900
20	autoclaved	P	cold	A	b	145000
27	autoclaved	P	cold	A	b	245000
34	autoclaved	P	cold	A	b	230000
41	autoclaved	P	cold	A	b	125000
47	autoclaved	P	cold	A	b	65000
50	autoclaved	P	cold	A	b	70800
54	autoclaved	P	cold	A	b	25600
90	autoclaved	P	cold	A	b	25400
113	autoclaved	P	cold	A	b	25600
148	autoclaved	P	cold	A	b	34900
0	autoclaved	P	cold	B	a	11000
1	autoclaved	P	cold	B	a	18000
4	autoclaved	P	cold	B	a	25200
8	autoclaved	P	cold	B	a	75200
20	autoclaved	P	cold	B	a	299000
27	autoclaved	P	cold	B	a	275000
34	autoclaved	P	cold	B	a	176000
41	autoclaved	P	cold	B	a	114000

47	autoclaved	P	cold	B	a	65000
50	autoclaved	P	cold	B	a	69100
54	autoclaved	P	cold	B	a	43300
90	autoclaved	P	cold	B	a	29700
113	autoclaved	P	cold	B	a	24700
148	autoclaved	P	cold	B	a	32400
0	autoclaved	P	cold	B	b	7000
1	autoclaved	P	cold	B	b	19000
4	autoclaved	P	cold	B	b	24300
8	autoclaved	P	cold	B	b	36500
20	autoclaved	P	cold	B	b	111000
27	autoclaved	P	cold	B	b	232000
34	autoclaved	P	cold	B	b	168000
41	autoclaved	P	cold	B	b	102000
47	autoclaved	P	cold	B	b	67000
50	autoclaved	P	cold	B	b	58500
54	autoclaved	P	cold	B	b	44000
90	autoclaved	P	cold	B	b	28000
113	autoclaved	P	cold	B	b	26700
148	autoclaved	P	cold	B	b	38700
0	autoclaved	R	warm	A	a	23000
1	autoclaved	R	warm	A	a	140800
4	autoclaved	R	warm	A	a	285000
8	autoclaved	R	warm	A	a	312000
20	autoclaved	R	warm	A	a	522000
27	autoclaved	R	warm	A	a	220000
34	autoclaved	R	warm	A	a	22000
41	autoclaved	R	warm	A	a	700
47	autoclaved	R	warm	A	a	120
50	autoclaved	R	warm	A	a	130
54	autoclaved	R	warm	A	a	140
57	autoclaved	R	warm	A	a	180
90	autoclaved	R	warm	A	a	450
99	autoclaved	R	warm	A	a	3460
113	autoclaved	R	warm	A	a	2690
148	autoclaved	R	warm	A	a	10
152	autoclaved	R	warm	A	a	50
0	autoclaved	R	warm	A	b	28000
1	autoclaved	R	warm	A	b	181800
4	autoclaved	R	warm	A	b	288000
8	autoclaved	R	warm	A	b	327000
20	autoclaved	R	warm	A	b	559000
27	autoclaved	R	warm	A	b	222000
34	autoclaved	R	warm	A	b	39000
41	autoclaved	R	warm	A	b	700
47	autoclaved	R	warm	A	b	190
50	autoclaved	R	warm	A	b	160

54	autoclaved	R	warm A	b	110
57	autoclaved	R	warm A	b	140
90	autoclaved	R	warm A	b	420
99	autoclaved	R	warm A	b	3640
113	autoclaved	R	warm A	b	2850
148	autoclaved	R	warm A	b	280
152	autoclaved	R	warm A	b	35
0	autoclaved	R	warm B	a	23000
1	autoclaved	R	warm B	a	153400
4	autoclaved	R	warm B	a	247000
8	autoclaved	R	warm B	a	260000
20	autoclaved	R	warm B	a	495000
27	autoclaved	R	warm B	a	172000
34	autoclaved	R	warm B	a	9000
41	autoclaved	R	warm B	a	300
47	autoclaved	R	warm B	a	150
50	autoclaved	R	warm B	a	150
54	autoclaved	R	warm B	a	330
57	autoclaved	R	warm B	a	270
90	autoclaved	R	warm B	a	2250
99	autoclaved	R	warm B	a	1630
113	autoclaved	R	warm B	a	1280
152	autoclaved	R	warm B	a	720
0	autoclaved	R	warm B	b	15000
1	autoclaved	R	warm B	b	121000
4	autoclaved	R	warm B	b	350000
8	autoclaved	R	warm B	b	268000
20	autoclaved	R	warm B	b	451000
27	autoclaved	R	warm B	b	219000
34	autoclaved	R	warm B	b	20000
41	autoclaved	R	warm B	b	300
47	autoclaved	R	warm B	b	140
50	autoclaved	R	warm B	b	200
54	autoclaved	R	warm B	b	300
57	autoclaved	R	warm B	b	420
90	autoclaved	R	warm B	b	1630
99	autoclaved	R	warm B	b	1900
113	autoclaved	R	warm B	b	1460
152	autoclaved	R	warm B	b	1055
0	autoclaved	D	warm A	a	3000
1	autoclaved	D	warm A	a	101000
4	autoclaved	D	warm A	a	254000
8	autoclaved	D	warm A	a	383000
20	autoclaved	D	warm A	a	128000
27	autoclaved	D	warm A	a	18000
34	autoclaved	D	warm A	a	3400
47	autoclaved	D	warm A	a	32100

50	autoclaved	D	warm A	a	23500
54	autoclaved	D	warm A	a	14800
57	autoclaved	D	warm A	a	8700
90	autoclaved	D	warm A	a	1800
113	autoclaved	D	warm A	a	6570
148	autoclaved	D	warm A	a	770
0	autoclaved	D	warm A	b	14000
1	autoclaved	D	warm A	b	114000
4	autoclaved	D	warm A	b	234000
8	autoclaved	D	warm A	b	367000
20	autoclaved	D	warm A	b	171000
27	autoclaved	D	warm A	b	19000
34	autoclaved	D	warm A	b	2600
47	autoclaved	D	warm A	b	31700
50	autoclaved	D	warm A	b	24300
54	autoclaved	D	warm A	b	1820
57	autoclaved	D	warm A	b	8100
90	autoclaved	D	warm A	b	2200
113	autoclaved	D	warm A	b	6360
148	autoclaved	D	warm A	b	810
0	autoclaved	D	warm B	a	13000
1	autoclaved	D	warm B	a	105000
4	autoclaved	D	warm B	a	280000
8	autoclaved	D	warm B	a	419000
20	autoclaved	D	warm B	a	119000
27	autoclaved	D	warm B	a	3000
34	autoclaved	D	warm B	a	400
0	autoclaved	D	warm B	b	7000
1	autoclaved	D	warm B	b	103000
4	autoclaved	D	warm B	b	259000
8	autoclaved	D	warm B	b	450000
20	autoclaved	D	warm B	b	94000
27	autoclaved	D	warm B	b	3000
34	autoclaved	D	warm B	b	700
0	autoclaved	P	warm A	a	8000
1	autoclaved	P	warm A	a	77000
4	autoclaved	P	warm A	a	426000
8	autoclaved	P	warm A	a	197000
20	autoclaved	P	warm A	a	108000
27	autoclaved	P	warm A	a	59000
34	autoclaved	P	warm A	a	14000
41	autoclaved	P	warm A	a	6400
47	autoclaved	P	warm A	a	2000
50	autoclaved	P	warm A	a	2530
54	autoclaved	P	warm A	a	2400
113	autoclaved	P	warm A	a	2100
148	autoclaved	P	warm A	a	5280

0	autoclaved	P	warm A	b	10000
1	autoclaved	P	warm A	b	79000
4	autoclaved	P	warm A	b	397000
8	autoclaved	P	warm A	b	182000
20	autoclaved	P	warm A	b	92000
27	autoclaved	P	warm A	b	68000
34	autoclaved	P	warm A	b	10000
41	autoclaved	P	warm A	b	9000
47	autoclaved	P	warm A	b	2200
50	autoclaved	P	warm A	b	1670
54	autoclaved	P	warm A	b	2100
113	autoclaved	P	warm A	b	900
148	autoclaved	P	warm A	b	3790
0	autoclaved	P	warm B	a	4000
1	autoclaved	P	warm B	a	80000
4	autoclaved	P	warm B	a	324000
8	autoclaved	P	warm B	a	190000
20	autoclaved	P	warm B	a	79000
27	autoclaved	P	warm B	a	59000
34	autoclaved	P	warm B	a	16000
41	autoclaved	P	warm B	a	6900
47	autoclaved	P	warm B	a	2500
50	autoclaved	P	warm B	a	2380
54	autoclaved	P	warm B	a	2860
90	autoclaved	P	warm B	a	7620
113	autoclaved	P	warm B	a	4500
152	autoclaved	P	warm B	a	15100
0	autoclaved	P	warm B	b	6000
1	autoclaved	P	warm B	b	67000
4	autoclaved	P	warm B	b	384000
8	autoclaved	P	warm B	b	151000
20	autoclaved	P	warm B	b	88000
27	autoclaved	P	warm B	b	72000
34	autoclaved	P	warm B	b	28000
41	autoclaved	P	warm B	b	7600
47	autoclaved	P	warm B	b	2500
50	autoclaved	P	warm B	b	2730
54	autoclaved	P	warm B	b	2750
90	autoclaved	P	warm B	b	10780
113	autoclaved	P	warm B	b	3100
152	autoclaved	P	warm B	b	28300
