# Supplementary material for: Die-off of plant pathogenic bacteria in tile drainage and anoxic water from a managed aquifer recharge site
Source: PLoS One. 2021 May 5;16(5):e0250338. doi: 10.1371/journal.pone.0250338 (PMC8099070; doi:10.1371/journal.pone.0250338)
Supplement: Attachment — Submitted filename: Response-to-the-reviewers.docx [file pone.0250338.s006.docx]

Response to the Editor

Ref:	PONE-D-20-35253
Title:	Die-off of plant pathogenic bacteria in tile drainage and anoxic water from a managed	aquifer recharge site
Journal:	PLOS ONE

Dear Dr. Eisfeld,

Thank you for submitting your manuscript to *PLOS ONE*. After careful consideration, we feel that it has merit but does not fully meet *PLOS ONE*’s publication criteria as it currently stands. Therefore, we invite you to submit a revised version of the manuscript that addresses the points raised during the review process.

Reply to the Editor:

Thank you for handling our manuscript and considering it for publication in *PLOS ONE*.

In the following, we address all comments raised by you and the reviewers and explain how we improved our paper to meet the publication criteria of *PLOS ONE*. Replies by the authors are indicated in pink, small textural changes are edited in the manuscript and are declared with “*Done.”* in this rebuttal letter. All changes can be followed up in the revised manuscript with track changes which was uploaded together with this rebuttal letter. Moreover, we adapted the funding statement according to *PLOS ONE*. The author with commercial affiliation was involved in the conceptualization and funding acquisition, but the funding did not influence the study design or any results.

**Specific comments by the Editor:**

1. In particular, criteria 3 and 4 (<https://journals.plos.org/plosone/s/criteria-for-publication>) have not been fully achieved. Both reviewers addressed the need to clarify experimental procedures, particularly related to the autoclaved dataset. The criterium to exclude this dataset is not clear, the explanation given in lines 345-347 “In the experiments with autoclaved water, in the initial stage, growth of the pathogens was observed. These data were not modelled, because autoclaved water is not representative of the actual natural conditions during MAR.” is vague. Why is the filtered TDW but not autoclaved TDW representative of the natural conditions?

Reply by the authors: We wanted to measure and simulate the die-off under natural conditions and know the effects of the naturally present microbiota. To investigate the latter we applied filtration or autoclaving, both widely used methods to remove/inactivate the naturally present microbiota. Filtration does not change the abiotic conditions whereas autoclaving does change the chemical composition. We observed temporal growth of the bacteria we added only under these conditions and hypothesized that this is likely due to the thermal breakdown of organic compounds in the autoclave, which made these compounds more readily biodegradable. As autoclaving does not represent natural abiotic conditions in comparison to the filtered microcosms, we did not model those results. In addition, the autoclaved results could demonstrate a new finding as *Dickeya solani* persisted in sterile autoclaved water for a prolonged time. This contrasts with the results of van Doorn et. al. (2008) who analyzed the survival of *Dickeya* spp. in sterile waters and reported that the bacteria rendered unculturable after a few days of incubation. To point this out, we added the following sentence after line 505 of the original manuscript:

“In addition, our results demonstrated that *D. solani* can persist in sterile autoclaved water for a prolonged time which seem to contrast with the findings of van Doorn et.al. [26] where *Dickeya* spp. rendered unculturable after a few days of incubation in sterile basin or ditchwater.”

Eventually, the autoclaved data serve as a control and we used them to compare results of natural and filtered treatments (line numbers refer to the original manuscript):

- line 396 et. seq.: “A similar observation was made in autoclaved TDW at 10 °C, where *R. solanacearum* is no longer culturable after a period of 30 days, even though the bacteria were culturable for a long period of 150 days at 25 °C (see S1 Fig).”
- line 421 et. seq.: “The faster die-off at 25 °C compared to 10 °C was noticeable in untreated and in filtered TDW, implying lower resistance of the SRP at a higher temperature. Still, the die-off in autoclaved TDW was similar at both temperatures.”
- line 436 et. seq.: “Note, that in all our experiments the bacteria were no more detectable by plating at the end of the observation periods. A persisting population was only observed in the autoclaved, artificial microcosms (see S1 Fig).”

The text passage in line 345-347 et. seq. (original manuscript) has been modified accordingly:

“Long-term persistence of the pathogens was demonstrated in the autoclaved microcosms where optimal conditions (available nutrients, no competition with other microorganism) resulted in growth of the pathogens in the beginning of the experiments. In the autoclaved microcosms the physicochemical composition of the water is changed during the heating process. In contrast, the filtration only removes the biotic fraction from the water leaves the abiotic conditions unchanged. This allows to study the influence of the biotic factors in oxic microcosms when comparing natural and 0.22 µm filtered TDW.”

2. Did authors measure the total and dissolved organic carbon in the natural and treated waters? Carbon content, particularly, dissolved organic carbon, would be an important parameter to consider in the die off of the bacteria, being a parameter affected particularly in the autoclaved treatment.

Reply by the authors: We did not measure total or dissolved organic carbon, but measured the chemical oxygen demand which represents the overall organic compounds in the water. Note, that COD also measures NH_4_, which was zero in the tested water samples (see Table 2 of the manuscript). The following sentence was added in the results section (line 352 of the original manuscript) regarding COD:

“The measured values of the chemical oxygen demand (COD) represent the total organic compounds in the water and they are representative for the range found in unpolluted surface waters (around 20 mg/L).”

3. Additionally, in the description of the treatments (line 212-226), it is not explained why the influence of the role of the natural microbial community in the anoxic natural aquifer was not evaluated. Why the authors did not consider it an important factor under these conditions?

Reply by the authors: The influence of the biotic fraction on the die-off of our target pathogens was shown under oxic conditions and therefore we considered it less relevant to demonstrate it also for anoxic water.

4. As pointed by the reviewers, it should be clearly stated by the authors that within the bacterial community only the culturable part was determined in the present study.

Reply by the authors:

We thank the editor and reviewers for their critical comments regarding this topic. We removed the sentence in line 193-194 (original manuscript) and added the missing information regarding the enumeration of indigenous culturable bacteria to a) 2.3 of material and methods (starting from line 219 of the original manuscript) and b) 2.4 of material and methods (line 274 of the original manuscript):

a)” For both the natural microcosms of oxic TDW and anoxic AW, a respective control microcosm that was not inoculated with pathogens was prepared.”

b) “The initial concentration of background culturable bacteria in the natural oxic and anoxic water samples was assessed by plating 0.1 mL on non-selective TSA and low-nutrient Reasoner’s 2A (R2A) agar (Oxoid; Thermo Fisher Scientific) and incubated at 25 °C. Anaerobic water samples were incubated anaerobically 25 °C using Oxoid AnaeroGen sachets (Oxoid; Thermo Fisher Scientific) to create an anaerobic atmosphere in a closed container. Additionally, total numbers of culturable bacteria were enumerated in all oxic TDW microcosms from both temperatures at the end of the die‑off period of the SRP.”

To be more specific on that topic, we added the following part to the results section in 3.1 (starting in line 339 of the original manuscript) and show the enumeration of culturable bacteria on TSA and R2A in S1 Table which we will add to the supplementary material.

“In natural TDW at 10 and 25 °C, the background culturable bacterial levels in the control microcosms were 10^2^ CFU/mL grown on TSA and R2A, and reached 9.6 – 9.8x10^3^ CFU/mL after 16 days, grown on TSA or R2A respectively. In the control microcosms of anoxic AW with or without nitrate, the concentration of culturable indigenous bacteria was about 2.3x10^2^ – 4x10^3^ CFU/mL, grown on TSA or R2A respectively. S1 Table shows an overview of the enumeration of culturable microorganism on TSA and R2A present in the native TDW and AW.”

Please note that S1 Table (containing information about the AIC values) provided in the initial submission will be renumbered to S2 Table.

5. Lines 64-66, please improve these sentences, and be careful when referring “we investigate the microbiological aspects of MAR”, as only the bacterial community was determined, and within this only the culturable members were determined;

Reply by the authors: The sentence in the introduction in lines 64-66 was modified: “In this study, we investigate the die‑off of bacterial plant pathogens in different water types from a MAR site to mimic the injection of contaminated water into the MAR system.”

6. In general, the English is good, the discussion is well written, but the abstract and results section needs to be improved. The text needs to be fluid and the abstract needs to be more concise and more assertive, so I advise a careful edition by someone whose first language is English.

Reply by the authors: We thank the editor for the positive comment about the discussion. The manuscript has been examined by a professional English editor. We shortened the abstract, specifically the first lines, and modified passages in the results section to be more concise and to improve readability. To see all changes, we refer to the revised manuscript with track changes.

7. Line 95, replace “D. solani and concentrations” by “D. solani at concentrations” Done.
8. Line 182, replace “Excess rainwater rain water reaches” by “Excess rainwater reaches”; Done.
9. Line 361-362, improve the sentence;

Reply by the authors: We made the following changes to clarify the sentence: “The experiments were concluded when the limit of detection of 3-10 CFU/mL was reached (visualized as horizontal dotted line in Figure 3) or the bacteria were no more detectable by plating (below the detection limit, as marked with an asterisk in Figure 3).”

10. Line 373-375, improve the sentence, is not clear;

Reply by the authors: We modified the sentence to make it more clear: “In anoxic AW, the addition of nitrate did not have a significant effect (P = 0.23) on the die-off of *R. solanacearum.”*

11. Line 400, “In addition, under anaerobic conditions the tailing effect is more pronounced (Fig 3, R5 & R6).”, is it? It seem that a longer tail is visible in R4 (filtered TDW, 25 ºC);

Reply by the authors: We agree that the longest tailing effect of the inactivation curve can be observed in Graph R4 of Figure 3 as it was also described in the manuscript. To avoid any misunderstanding, we modified the sentence by combining the two sentences:

“In filtered TDW at 25 °C, the bacteria persisted at a low concentration of ca. 10 CFU/mL for up to 60 days creating a long tail‑shape in the plotted curve (see Fig 3, R4), which can also be observed under anaerobic conditions for a period of ca. 25 days (Fig 3, R5 & R6). Surprisingly, the die-off of *R. solanacearum* under anoxic conditions took about 45 days and was two times slower than in natural TDW despite the low temperature of 10 °C and absence of oxygen.”

12. Line 402-406, these two sentences can be combined, in a simpler sentence;

Reply by the authors: We changed the sentences to ease readability, see also comment 11: “Surprisingly, the die-off of *R. solanacearum* under anoxic conditions took about 45 days and was two times slower than in natural TDW regardless the low temperature of 10 °C and absence of oxygen.”

13. Line 440, replace “Graph R5…” by “The fitting plotted in R5 and R6”; Done.
14. Line 441-443, replace “In the graph, R5 has the longest shoulder (β = 5.63) where the initial population stays constant for nearly 20 days. In contrast, the shoulder in R6 is observed during the first 15 days expressed in a smaller β of 3.1.” by “ In fact, R5 has the longest shoulder where the initial population stays constant for nearly 20 days (β = 5.63). In contrast, the shoulder in R6 is a smaller (β = 3.1).”; Done.
15. Line 554, replace “As a result, this flexibility” by “As a result, the flexibility”. Done.

Additional modification of the manuscript by the authors: With outlook to future risk modelling, we added two sentences after line 578 (original manuscript) to avoid misunderstandings by the reader.

“Therefore, the *C_res_* parameter should not be handled as an absolute value, but as a fraction of the initial *C_0_* concentration in risk modelling. Otherwise, the final concentration after treatment of even low (> 10 CFU/mL) contaminated water would mathematically always result in *C_res_* regardless of the initial *C_0_.”*

**Reply to Reviewer 1:**

Reviewer #1: Summary

With increasing concerns for shortage of fresh water for agriculture, managed aquifer recharge (MAR) practice that stores excess fresh water, such as tile drainage water (TDW), offers a valid source of irrigation water for crops. Unfortunately, the water can be contaminated and be responsible for dissemination of plant pathogens upon irrigation. In order to understand and develop a model to estimate the survival and persistence of plant pathogens in TDW and AW (aquifer water), the authors determined the die-off rate of three plant pathogens as a function of oxygen, temperature, and resident microbiota. They developed a flexible non-linear Weibull model to predict bacterial die-off to determine the persistence of the plant pathogens in TDW and AW. Although each bacterial pathogen behaves differently, the developed model can be used as a part of an initial pathogen risk assessment to determine the potential safety of the stored water for safe irrigation of agricultural crop. It is hoped that with the model, they can develop a safe approach to remove the potential pathogens from the stored water.

Although there are some concerns with the experimental procedures, the authors were largely successful in developing a non-linear model to predict the survivability and persistence of three plant pathogens as a function of oxygen, temperature, and the resident microbiota.

**Specific comments by reviewer 1:**

1. Most of the major concerns were with microbiological experiments.

   How were the indigenous microbiota determined? Based on the description on page 8, lines 193-194, it is unclear whether TSA or R2A agar were used for both oxic and anoxic water samples and how the plates were incubated. One would hope that the anoxic water sample containing plates were incubated under anaerobic conditions in order to determine the anaerobes present in the sample but it is not clear from their description. Furthermore, the incubation temperature is also important to grow organisms that have adapted to colder temperature. Another important aspect that should have been taken into account for both indigenous microbes as well as inoculated plant pathogens after a prolonged incubation in the water samples is to have plated them on nutrient poor media. The authors used R2A but it is not clear from their description, exactly which samples were plated on TSA versus R2A.

Reply by the authors: We thank the reviewer for the critical comment to improve our manuscript and refer to editor’s comment #4 for the reply of the authors.

2. Finally, given that less than 1% of the bacteria in nature can be cultured, the authors SHOULD have performed metagenomic analysis to determine the indigenous microbiota in these water samples because they discuss potential competition by these organisms on plant pathogens throughout the manuscript. A better understanding of the indigenous microbiota and their effect on pathogens will also help understand the pathogen persistence in water from different locations which would have to be performed to validate the model.

Reply by the authors: Metagenomic analysis could indeed give insight in to the microbiome in the water types, but not about their activity nor about direct interactions with the studied pathogens. We considered the application of metagenomics techniques, but decided that they would not have aided in explaining the established die‑off kinetics which is the scope of this paper. Further investigations on interactions between target pathogens and the microbiome (activity) are of interest but should be addressed in future research.

To clarify that we only analyzed culturable microorganisms, we added the following sentences after line 512 (of the original manuscript) to the discussion for clarification: “In this study, the background microbiota was only characterized by plating TSA and R2A medium at 25 °C. Further research is required to elucidate any specific interactions between the examined pathogens and their environment including other microorganisms in the microbiome.”

To point it out again, we also added a sentence at the end of 4.3 of the discussion (line 538 of the original manuscript): “This study focused on the die‑off kinetics of the pathogens and only analyzed the culturable part of the indigenous microbiota. Interactions between the pathogens and specific microorganisms or bacteriophages may affect the die-off kinetics, but this is topic for future research. “

3. Since the authors did not use the autoclaved water data in their modeling because of the initial growth of the inoculated bacteria (likely due to the dead indigenous organisms serving as nutrients), it is unclear whether discussion of this condition serves any purpose for this manuscript, even as supplemental data.

Reply by the authors: Thank you for the comment. Please see our reply on editor’s comment #1 regarding this topic.

4. Although it may be out of scope for this particular manuscript, it would have been helpful to have addressed the relative metabolic change of the three bacteria as a function of temperature and oxygen. That may help explain the observed data especially with regards to the temperature which the authors call "temperature sensitivity" on page 23, line 394.

Reply by the authors: We agree with the reviewer, that the observation of the metabolic changes could have been an interesting aspect to observe. But as the reviewer already mentions, this was out of scope for this study. Moreover, it may be difficult to differentiate between the metabolic state of the target pathogen within a water microcosm where also other microorganism are present.

5. Figure 3 needs to be improved to clearly show the data. Unlike other figures in the manuscript, this figure was fuzzy and out of focus.

Reply by the authors: It was possible to download the figure separately. In the composed PDF, the figure is indeed blurry, but *PLOS ONE* offers the possibility to download the picture where it can be received with sufficient good quality (see also: <https://www.editorialmanager.com/pone/download.aspx?id=27777079&guid=e3003e13-1a2e-4a23-b6c6-98cc59193cb6&scheme=1>).

6. There were some spelling errors that should be corrected.
7. This is not a concern but a positive critique. The Discussion was well-written and addressed most of the concerns of the data in the manuscript with the exception of those addressed above.

**Specific comments by reviewer 2:**

Reviewer #2: GENERAL COMMENTS:
This manuscript investigated the die-off of three important plant pathogens, Ralstonia solanacearum, Dickeya solani, and Pectobacterium carotovorum sp. carotovorum in natural tile drainage water and anoxic aquifer water under different conditions. The collected data was used to fit the best die-off model by comparing a non-linear Weibull+ tail, a Weibull, and a modified log-linear model in order to predict the fate of these pathogens for risk assessments in recycling water systems. This manuscript is worthy of publication in PLOS One following major revision in terms of its applicable value. However, there are issues in the manuscript that need to be clarified and revised before publishing.

Reply by the authors: We thank the reviewer to consider our manuscript for publication in *PLOS ONE* and for the comments to improve our manuscript.

1. Suggestions for improvement of the manuscript are outlined below.
   Pg2, Ln35, Ln82: change ‘*Pectobacteriaceae’* to ‘Pectobacteriaceae’. Family name should not be italic. This occurred in several places across the manuscript, so please address it. Done.
2. Pg2, Ln64-66: consider deleting the two sentences, or move them to the last paragraph of the introduction. Done, also according to editor, comment #5.
3. Pg3, Ln68: consider adding ‘also known as brown rot’ after ‘They can cause bacterial wilt’. Your audience (without a background in plant pathology) may be confused when you mention potato brown rot in Ln72. Done.
4. Pg4, Ln85-86: suggest deleting “For example… glasshouse ornamental crops”. This is a redundant sentence. You have mentioned its broad host range in Ln69.

Reply by the authors: As this papers covers an interdisciplinary topic, we would like to keep this sentence to address a broader audience from different backgrounds. Readers with an engineering background who are not familiar with plant pathogens might not think about flower bulbs as hosts when initially talking about potato pathogens.

5. Pg4, Ln87-97: Please consider merging this paragraph with the previous paragraph. Done.
6. Pg4, Ln93: Change ‘(e.g. [16])’ to ‘[16]’. Done.
7. Pg4, Ln94-95: consider changing to ‘Kastelein et al. [17] reported that spray inoculation of potato leaves with 102 CFU/mL D. solani resulted in diseased plants in greenhouse experiments.’ Done.
8. Pg4, Ln95-97: consider deleting ‘Moreover… potato material’. You have mentioned SRP can be found in the environment in Ln91. Done.
9. Pg5, Ln117: change ‘, as well as the natural microbiota,’ to ‘and the natural microbiota’. Done.
10. Pg5, Ln119: change ‘No study, however, was found that describes’ to ‘However, no study has reported that the die-off…’. Done.
11. Pg6, Ln154: change ‘Streptomycin’ to ‘streptomycin’. Done, also corrected in other parts of the manuscript, see also comment #20.
12. Pg7, Ln 163-164: change ‘the aquifer storage… (ASTR) system’ to ‘The ASTR system’; change ‘tile drainage water (TDW)’ to ‘TDW’. This also applies to other places across the manuscript. Done.
13. Pg7, Ln166: delete ‘Figure 1 shows a scheme of the ASTR system’, and add ‘, as shown in Figure 1,’ right after ‘The ASTR system’ in Ln164. Done.
14. Pg8, Ln182: delete ‘rain water’. Done.
15. Pg8, Ln186: add ‘.’ after ‘irrigation’. Done.
16. Pg8, Ln193-194: please clarify this sentence. You can only assess culturable microbes by plating on medium. The plating method cannot represent the microbiota. This issue occurred in several places across the manuscript. Please address them as well. In addition, please spell out R2A agar as this is the first time it appeared in the text.

Reply by the authors: Thank you for your comment. For the reply we refer to reviewer 1’s comment #1.

17. Pg11, Ln256-258: rewrite these two sentences and add references.

Reply by the authors: The sentences have been modified and shortened and the reference placed accordingly: “Wenneker et. al. [10] detected *R. solanacearum* in ditches next to agricultural fields at concentrations ranging between 10^2^ – 10^5^ CFU/mL. Higher inoculum density may give the introduced pathogenic bacteria a competitive advantage against other microorganisms.”

18. Pg11, Ln261: change ‘, plus’ to ‘and’. Done.
19. Pg11, ln268: spell out SMSA. Done.
20. Pg11, Ln269, Ln271: change ‘Rifampicin’ to ‘rifampicin’; change ‘Streptomycin’ to ‘streptomycin’. This occurred in several other places, please address them in order to be consistent. Done, see also comment #11.
21. Pg11-12, Ln272-274: add reference(s).

Reply by the authors: To select the best medium with the highest recovery rate and best suppression of other microorganisms, we did a small preliminary test, where a known concentration of bacteria was added to tile drainage water and recovered by plating. To clarify, we modified the sentence:

“The selective media have been chosen as they showed a higher recovery rate from the environmental water samples in comparison to TSA supplemented with the respective antibiotic (unpublished results).”

22. Pg12, Ln 274-276: Again, you can only determine culturable bacteria through TSA or R2A. It is not correct to use the term ‘microbiota’ since you only measured culturable bacteria.

Reply by the authors: Thank you for the comment, we edited the sentence and replaced the term ‘microbiota by ‘culturable bacteria’.

23. Pg12, Ln278: how many replicates? Done.
24. Pg12, Ln283L: does ‘the bacterial concentration’ here refer to the culturable bacteria or the tested three phytopathogens?

Reply by the authors: For clarification, we modified the sentence to: “… the bacterial concentration defined as culturable cells per mL… “

25. Pg13, Ln318-320: suggest changing to ‘All three models were applied to fit the experimental data and the best model was chosen based on the Akaike Information Criterion (AIC)’. Done.
26. Pg13, Ln320-325: suggest deleting the description on AIC (this is commonly known, and you have directed your audience to the reference as well).

Reply by the authors: We agree with the comment of the reviewer and shortened the paragraph about the AIC to the following: “The AIC considers the goodness of fit and parsimony of the models. For our experiments, the model with the smallest AIC value was therefore chosen as best model.”

27. Pg15, Ln339: change ‘microbiota’ to ‘culturable bacterial’. Done.
28. Pg15, Ln340: what is the SD of the background bacterial concentration (102 -104 CFU/mL)?

Reply by the authors: We added detailed information about the background bacterial concentrations in the S1 Table, which will be included to the supplementary material. We also refer to editor’s comment #4 and our reply on this topic.

29. Pg15, Ln350: delete the comma between ‘modelling’ and ‘because’. This also occurred in other places, please address them. Done.
30. Pg16, Ln366: add ‘both’ before ‘selected’. Done.
31. Pg16, Ln370-371 ‘The results of experiments… in S1 Fig’, please at least briefly describe your results of the autoclaved experiments here.

Reply by the authors: We added a brief description after the sentence: “There, the bacteria started to grow at 10 and 25 °C reaching a concentration of 10^6^ CFU/mL followed by a slow decline until they reached a concentration of at least 10^3^ CFU/mL which lasted until the end of the observation period (150 days). The only exception from this trend was the die‑off of *R. solanacearum* in the autoclaved TDW at 10 °C which rendered non‑culturable after a period of approximately 30 days.”

32. Pg16, Ln377: add ‘was’ before ‘slower’. Done.
33. Pg18-19, ln439-440: delete ‘, which will be discussed later.’ Done.
34. Pg21, Ln465: delete ‘state’. Done.
35. Pg21, Ln471: delete ‘,’. Done.
36. Pg22, Ln493: delete ‘;’. Done.
37. Pg22-23, session 4.3: Is there a difference between culturable bacteria in the background in between TDW and AW? Where is the data?? Please see comment #28, the full data has now been added as S1 Table.
38. Pg24, Ln541: change ‘no more’ to ‘not’. Done.
39. Pg25, Ln563-564: change ‘On the other hand’ to ‘In addition’. The sentence before, starts with ‘On the one hand’, hence the second expression ‘On the other hand’ needs to remain.
40. Pg26, Ln 604: change ‘can be’ to ‘could be’ Done.
41. Pg26, Ln 606-607: reconsider the statement: ‘no risk inducing plant diseases.’ It may be the fact, but I would not say without a supporting data or listing a reference.

Reply by the authors: This comment is meant as a future outlook therefore the sentence contains the expression “in order to” and should not be treated as a final assumption.

42. Pg28, reference session: font and size are different compared to previous sessions. The font and size of the references have been adapted to the rest of the manuscript.
