# Supplementary material for: Die-off of plant pathogenic bacteria in tile drainage and anoxic water from a managed aquifer recharge site
Source: PLoS One. 2021 May 5;16(5):e0250338. doi: 10.1371/journal.pone.0250338 (PMC8099070; doi:10.1371/journal.pone.0250338)
Supplement: Attachment — Submitted filename: Response to Reviewers.docx [file pone.0250338.s007.docx]

Response to Reviewers

PONE-D-20-35253R1
Die-off of plant pathogenic bacteria in tile drainage and anoxic water from a managed aquifer recharge site
PLOS ONE

Dear Dr. Eisfeld,

Thank you for submitting your manuscript to PLOS ONE. After careful consideration, we feel that it has merit but does not fully meet PLOS ONE’s publication criteria as it currently stands. Therefore, we invite you to submit a revised version of the manuscript that addresses the points raised during the review process.

Although the authors have addressed all the previous suggestions, there are still some points that need to be addressed.

**Reply by the authors:**

We thank the editor for the second revision of our manuscript and giving useful comments to improve our paper for publication in *PLOS ONE*. We are pleased that our first revision answered the questions of the Editor and the Reviewers satisfactorily. Hereby, we would like to address the remaining questions. Replies by the authors are indicated in pink and small textural changes are edited in the manuscript and are declared with “*Done”* in this rebuttal letter. All changes in the revised manuscript were made using the track changes function in MS Word. It was uploaded together with this rebuttal letter.

Regarding the culturable indigenous bacteria present in both oxic and anoxic microcosms, the inclusion of table S1 was important. But the duration of the experiments is not clear, based on the days presented in Fig 3 and on table S1. Enumerations in the controls are presented for day 0 and 16, but not all the experiments ended at day 16, right?

**Response by the authors:** The enumerations were done at day 0, 12 and 16 post inoculation (dpi) and indeed, not all experiments were concluded at the time point of 16 dpi. The duration of the experiments can be followed in the die-off curves of Figure 3. An exact duration of the sampling of each experiment can be found in the Supplementary File 2 and 3. For clarification, we added the following sentence to Table S1:

“*Controls are non-inoculated water microcosm and the time indication refers to the start of the experiment when pathogen microcosms were inoculated. Data on the total duration of the die‑off experiments are found in Figure 3.”

Besides, for the inoculated microcosms there is no enumeration of the culturable bacteria at day 0, and also the enumeration at the end of the experiment is missing. The experiments were longer than 12 or 16 days, right?

**Response by the authors:** The anoxic and oxic control microcosms were prepared with the same water type on the same day as the inoculated ones. Therefore, the inoculated microcosms were not tested at day 0 because they will have the same concentration in culturable bacteria as the controls. After inoculation at day 0, the concentration of culturable bacteria was increased by 10^4^ CFU/mL which refers to the concentration of introduced pathogen. The initial pathogen concentration was only evaluated on day 0 with the semi-selective media supplemented with antibiotics in which the growth of indigenous bacteria was suppressed. No enumeration of culturable bacteria was done at the end of the experiment. Nevertheless, the scope of this study was the die‑off kinetics and not the specific influence of indigenous microorganism on the pathogens. Enumeration of the culturable bacteria allows to determine the potential effect of the indigenous microbiome on the pathogens, although we realize that this will also depend on the composition of the microbiome.

Besides, in the material and methods the authors referred line 213-214 “Experiments with natural aquifer water where nitrate was added, were only tested with R. solanacearum and D. solani.”, why not evaluating the die-off of *P. carotovorum* sp. *carotovorum* under this condition?

**Response by the authors:** *D. solani* and *P. carotovorum* sp. *carotovorum* both belong to the family of the soft rot Pectobacteriaceae (SRP). After observing that their die-off of in oxic natural or filtered tile drainage water (TDW) was very similar, we decided to only test one of the SRP for the effect of nitrate. In addition, only a small effect one die‑off of the *D. solani* or *R. solanacearum* was found and therefore we decided to not further extend investigations on this topic.

Line 165-170, why is this information important?

**Response by the authors:** During managed aquifer recharge, oxic TDW is infiltrated into an anoxic aquifer which may change the hydrochemistry of the aquifer over a long-term as well as the microbial community of the aquifer. With the information in line 165-170 we want to inform the reader that the anoxic water sample was derived from a relatively (1 year) undisturbed aquifer where a stable hydrochemistry and aquifer community can be found.

Small issues that need to be addressed:

Abstract

Line 31 Delete “0.22 μm”; **Done.**

Line 31 replace “anoxic aquifer water” by “anoxic aquifer water (AW)”; **Done.**

Line 32 Replace “are” by “were”; **Done.**

Line 40-41 remove “Higher temperature and the presence of background microbiota strongly

41 accelerated die-off.”; **Done.**

Line 41, replace “In anoxic natural aquifer” by “Whereas In anoxic natural aquifer”; **Done.**

Line 44-46, replace “The non-linear model provides a tool to reliably predict the die-off of plant pathogenic bacteria or other pathogenic microorganisms in the context of microbial risk assessment.”

by “The non-linear model was shown to be a reliable tool to predict the die-off of the analyzed plant pathogenic bacteria, suggesting its further application to other pathogenic microorganisms in the context of microbial risk assessment.”; **Done.**

Material and Methods

Table 1, move the row “treatment” to the top of the table, above the row water type; **Done.**

Line 209-211, replace “For both the natural microcosms of oxic TDW and anoxic AW, a respective control microcosm that was not inoculated with pathogens was prepared” by “For both the natural microcosms of oxic TDW and anoxic AW a non-inoculated control microcosm was prepared. **Done.**

Line 214-218, replace “The temperatures were chosen as they represent, at the lower end, the nearly constant temperature in the aquifer (10 °C); while the upper end (25 °C) reflects infiltration of rainfall events during the warmer summer months. Moreover, it covers a temperature that is more representative for tropical regions, where these bacteria also play an important role as disease causing organisms.”

By “The temperatures were chosen as they represent the nearly constant temperature in the aquifer (10 °C); while 25 °C reflects infiltration of rainfall events during the warmer summer months. Moreover, the last covers a temperature more representative of tropical regions, where these bacteria also play an important role as disease causing organisms.”; **Done.**

Line 220, replace “microbiome” by “microbial”; **Done.**

Line 221, remove “a sterile filter holder with receiver (Nalgene, USA) and”; **Done.**

Line 265, replace “unpublished results” by “data not shown”; **Done.**

Line 266/511/, replace “background” by “indigenous”; **Done.**

Line 279, replace by “the bacterial” by “the pathogen”; **Done.**

Figure 2, replace by “the population concentration” by “the pathogen concentration”; **Done.**

Results

Line 363/367, replace “S1 Table” by “Table S2”; **Done.**

Line 370, replace “in S1 Fig” by “Fig S1”; **Done.**

Line 443, replace “faster resulting in a higher α of 0.39, and β is 2.68 producing a two times shorter shoulder phase” by “faster resulting in a higher α of 0.39 a lower β of 2.68 producing a two times shorter shoulder phase than at 10 °C”; **Done.**

Discussion

Line 462, replace “not further analyzed” by “not evaluated”; **Done.**

Line 480-482, replace “Therefore, our study is the first to analyze the die-off of R. solanacearum phylotype II under anoxic conditions in natural water, and further investigation should elucidate the metabolic pathway to these conditions.” by ““Therefore, our study is the first to analyze the die-off of R. solanacearum phylotype II under anoxic conditions in natural water. Further investigation should elucidate the metabolic activity of R. solanacearum under these conditions.”; **Done.**

Line 496, replace “at 25 and 10 °C” by “at 25 than at 10 °C”; **Done.**

Line 496, replace “For the SRP, we also observed a faster decline within 4 days at a higher” by “Whereas for the SRP, we observed a faster decline within 4 days at a higher”; **Done.**

Line 499-500, replace “The long-term persistence in sterile aquatic microcosms, unaffected by the present microbiota, has been shown earlier for the SRP” by “The long-term persistence in sterile aquatic microcosms, where the indigenous microbiota was absent, has been shown earlier for the SRP”; **Done.**

Line 505, replace “that it is better adapted” by “that the former is better adapted”; **Done.**

Line 510, replace “against the microbiota” by “against the existing microbiota”; **Done.**

Line 511-512, replace “microbiota was only characterized by plating on TSA and R2A medium at 25 °C” by “microbiota was only represented by the culturable community (growing in TSA and R2A, respectively).”; **Done.**

Line 513, replace “the microbiome” by “microbial community”; **Done.**

Line 518-519, replace “Interestingly, we observed similar die-off periods in anoxic AW for all the tested pathogens,” by “Interestingly, we observed similar and longer die-off periods in anoxic Aw than in TDW for all the tested pathogens.”; **Done.**

Line 519-521, replace “even though the culturable concentrations of present background bacteria were comparable in the TDW and AW. Nevertheless, the composition of the microbiota might differ in the AW and TDW, as reported before [52].” by “Although TDW and AW presented comparable concentrations of culturable bacteria the composition of the microbiota might differ between both, as previously reported [52].”; **Done.**

Line 521-523, replace “There, the authors state that the total diversity of the microbiota in shallow aquifers is lower than in the overlying surface, which supports the different die-off periods of the pathogens in natural TDW and AW, influenced by the present microbiota. The TDW used in our…” by “These authors stated that the total diversity of the microbiota in shallow aquifers is lower than in the overlying surface, which could support the different die-off periods of the pathogens in natural TDW and AW, observed in the present study. In fact, the TDW used in our…” **Done.**

Line 527, replace “The effects of the different fractions of the native microbiota on the pathogens have not been further explored in this study, but were subjected for R. solanacearum by Alvarez et. al. [22]. There, the native microorganisms altogether (protozoa, bacteria,…” by “Although, the effects of the different fractions of the native microbiota on the pathogens were not accessed in this study, previous studies reported that the native microorganisms (e.g., protozoa, bacteria,…” **Done.**

Line 534, replace “persistence of the pathogens” by “persistence of the pathogens, under this condition”; **Done.**

Line 536, replace “products” by “changes”; **Done.**

Line 537, replace “as well” by “and to improve”; **Done.**

Line 537-540, replace “To conclude, the introduced pathogens competed better against the AW microbiota than of the TDW, where fewer predators (e.g. protozoa) are present, or where the absence of oxygen can change the metabolic responses of the bacteria which increase their fitness in the AW.” by “The obtained data suggests that the inoculated pathogens competed better against the AW microbiota than to the TDW, where fewer predators (e.g. protozoa) are present, or where the absence of oxygen induces metabolic responses, increasing the pathogen fitness in the AW.”; **Done.**

Line 542-543, replace “, but this is topic for future research.” by “and needs to be further addressed.” **Done.**

Line 584, replace “>” by “<”; **Done.**

Conclusions

Line 618, remove “from”; **Done.**

Line 619, replace “took between” by “occurred between”; **Done.**

Line 624-625, replace “increases which will especially affect agricultural production that accounts for about 70% of fresh water use.” by “increases, which will affect especially agricultural production accounting for about 70% of fresh water use”; **Done.**

Line 625-627, replace “MAR is an option to store fresh TDW in the subsurface for later use as irrigation water but water quality aspects need to be considered as (plant) pathogens may enter the system.” by “MAR is an option to store fresh TDW in the subsurface for later use as irrigation water but water quality, particularly, related to the presence of plant pathogens needs to be considered.” **Done.**

Please submit your revised manuscript by Apr 16 2021 11:59PM. If you will need more time than this to complete your revisions, please reply to this message or contact the journal office at plosone@plos.org. When you're ready to submit your revision, log on to https://www.editorialmanager.com/pone/ and select the 'Submissions Needing Revision' folder to locate your manuscript file.

Please include the following items when submitting your revised manuscript:

- A rebuttal letter that responds to each point raised by the academic editor and reviewer(s). You should upload this letter as a separate file labeled 'Response to Reviewers'.
- A marked-up copy of your manuscript that highlights changes made to the original version. You should upload this as a separate file labeled 'Revised Manuscript with Track Changes'.
- An unmarked version of your revised paper without tracked changes. You should upload this as a separate file labeled 'Manuscript'.

If you would like to make changes to your financial disclosure, please include your updated statement in your cover letter. Guidelines for resubmitting your figure files are available below the reviewer comments at the end of this letter.

If applicable, we recommend that you deposit your laboratory protocols in protocols.io to enhance the reproducibility of your results. Protocols.io assigns your protocol its own identifier (DOI) so that it can be cited independently in the future. For instructions see: <http://journals.plos.org/plosone/s/submission-guidelines#loc-laboratory-protocols>

We look forward to receiving your revised manuscript.

Kind regards,

Ana R. Lopes, PhD
Academic Editor
PLOS ONE


[Note: HTML markup is below. Please do not edit.]

Reviewers' comments:

Reviewer's Responses to Questions

**Comments to the Author**

1. If the authors have adequately addressed your comments raised in a previous round of review and you feel that this manuscript is now acceptable for publication, you may indicate that here to bypass the “Comments to the Author” section, enter your conflict of interest statement in the “Confidential to Editor” section, and submit your "Accept" recommendation.

Reviewer #2: All comments have been addressed

2. Is the manuscript technically sound, and do the data support the conclusions?

The manuscript must describe a technically sound piece of scientific research with data that supports the conclusions. Experiments must have been conducted rigorously, with appropriate controls, replication, and sample sizes. The conclusions must be drawn appropriately based on the data presented.

Reviewer #2: Yes

3. Has the statistical analysis been performed appropriately and rigorously?

Reviewer #2: Yes

4. Have the authors made all data underlying the findings in their manuscript fully available?

The [PLOS Data policy](http://www.plosone.org/static/policies.action#sharing) requires authors to make all data underlying the findings described in their manuscript fully available without restriction, with rare exception (please refer to the Data Availability Statement in the manuscript PDF file). The data should be provided as part of the manuscript or its supporting information, or deposited to a public repository. For example, in addition to summary statistics, the data points behind means, medians and variance measures should be available. If there are restrictions on publicly sharing data—e.g. participant privacy or use of data from a third party—those must be specified.

Reviewer #2: Yes

5. Is the manuscript presented in an intelligible fashion and written in standard English?

PLOS ONE does not copyedit accepted manuscripts, so the language in submitted articles must be clear, correct, and unambiguous. Any typographical or grammatical errors should be corrected at revision, so please note any specific errors here.

Reviewer #2: Yes

6. Review Comments to the Author

Please use the space provided to explain your answers to the questions above. You may also include additional comments for the author, including concerns about dual publication, research ethics, or publication ethics. (Please upload your review as an attachment if it exceeds 20,000 characters)

Reviewer #2: The updated version has been largely improved, and the authors have addressed my previous concerns. Good work.

7. PLOS authors have the option to publish the peer review history of their article ([what does this mean?](https://journals.plos.org/plosone/s/editorial-and-peer-review-process#loc-peer-review-history)). If published, this will include your full peer review and any attached files.


If you choose “no”, your identity will remain anonymous but your review may still be made public.


**Do you want your identity to be public for this peer review?** For information about this choice, including consent withdrawal, please see our [Privacy Policy](https://www.plos.org/privacy-policy).

Reviewer #2: No

[NOTE: If reviewer comments were submitted as an attachment file, they will be attached to this email and accessible via the submission site. Please log into your account, locate the manuscript record, and check for the action link "View Attachments". If this link does not appear, there are no attachment files.]

While revising your submission, please upload your figure files to the Preflight Analysis and Conversion Engine (PACE) digital diagnostic tool, <https://pacev2.apexcovantage.com/>. PACE helps ensure that figures meet PLOS requirements. To use PACE, you must first register as a user. Registration is free. Then, login and navigate to the UPLOAD tab, where you will find detailed instructions on how to use the tool. If you encounter any issues or have any questions when using PACE, please email PLOS at [figures@plos.org](mailto:figures@plos.org). Please note that Supporting Information files do not need this step.
